# Allele balance bias identifies systematic genotyping errors and false disease associations

**DOI:** 10.1002/humu.23674

**Published:** 2018-11-23

**Authors:** Francesc Muyas, Mattia Bosio, Anna Puig, Hana Susak, Laura Domènech, Georgia Escaramis, Luis Zapata, German Demidov, Xavier Estivill, Raquel Rabionet, Stephan Ossowski

**Affiliations:** ^1^ Centre for Genomic Regulation (CRG) The Barcelona Institute of Science and Technology Barcelona Spain; ^2^ Universitat Pompeu Fabra (UPF) Barcelona Spain; ^3^ Institute of Medical Genetics and Applied Genomics University of Tübingen Tübingen Germany; ^4^ CIBER in Epidemiology and Public Health (CIBERESP) Barcelona Spain; ^5^ Sidra Medicine Doha Qatar; ^6^ Women's Health Dexeus Barcelona Spain; ^7^ Institut de Recerca Sant Joan de Déu; Institut de Biomedicina de la Universitat de Barcelona (IBUB) ; & Department of Genetics Microbiology and Statistics University of Barcelona Barcelona Spain

**Keywords:** allele balance, false positive variant calls, genetic variant detection, systematic NGS errors

## Abstract

In recent years, next‐generation sequencing (NGS) has become a cornerstone of clinical genetics and diagnostics. Many clinical applications require high precision, especially if rare events such as somatic mutations in cancer or genetic variants causing rare diseases need to be identified. Although random sequencing errors can be modeled statistically and deep sequencing minimizes their impact, systematic errors remain a problem even at high depth of coverage. Understanding their source is crucial to increase precision of clinical NGS applications. In this work, we studied the relation between recurrent biases in allele balance (AB), systematic errors, and false positive variant calls across a large cohort of human samples analyzed by whole exome sequencing (WES). We have modeled the AB distribution for biallelic genotypes in 987 WES samples in order to identify positions recurrently deviating significantly from the expectation, a phenomenon we termed allele balance bias (ABB). Furthermore, we have developed a genotype callability score based on ABB for all positions of the human exome, which detects false positive variant calls that passed state‐of‐the‐art filters. Finally, we demonstrate the use of ABB for detection of false associations proposed by rare variant association studies. Availability: https://github.com/Francesc-Muyas/ABB.

## 1 INTRODUCTION

The rapid improvement of next‐generation sequencing (NGS) throughput and cost has changed biomedical research as well as clinical diagnostics of genetic diseases and cancer (Altmann et al., 2012). Numerous genome‐sequencing projects catalogued millions of frequent and rare variants, some of which are associated to disease (Auton et al., 2015). NGS has facilitated the identification of novel therapeutic targets or genomic markers for clinical diagnostics and treatment, becoming the technology of choice to study the genetic causes of diseases (Hwang et al., 2015; Oleksiewicz et al., 2015; Pabinger et al., 2014).

Despite the widespread use of NGS in genetic disease studies and diagnostics, the use of short reads for identification of causal or disease‐associated variants is still sensitive to technical errors and may generate false associations and diagnoses (Hardwick, Deveson, & Mercer, 2017; Lee, Abecasis, Boehnke, & Lin, 2014; Yan et al., 2016). If the studied event is rare, such as *de novo* germline mutations, the likelihood to observe false positive (FP) calls is further increased (Gómez‐Romero et al., 2018; Veltman & Brunner, 2012). Moreover, rare variant association studies (RVAS) can generate false results if genes are enriched with sequencing or alignment errors, leading to false associations to the studied disease (Hou et al., 2017; Johnston, Hu, & Cutler, 2015; Yan et al., 2016). Hence, some RVAS methods take into account error probabilities (He et al., 2015) or bypass genotype calls completely by directly modeling sequencing reads (Hu, Liao, Johnston, Allen, & Satten, 2016). The impact of false genotype calls is amplified in the study of recurrent somatic mutations in cancer, particularly, if ultra‐deep sequencing is used to identify sub‐clonal mutations with low minor allele frequency (Cai, Yuan, Zhang, He, & Chou, 2016). Moreover, recent benchmarking studies reported substantial disagreement between somatic SNV and indel prediction methods (Alioto et al., 2015).

Although most variant calling algorithms can deal with random sequencing errors, systematic errors have mostly been neglected in the past and thus more often lead to FP variant calls. Several causes of errors of sequencing by synthesis‐based platforms are well described, such as *crosstalk* and *dephasing* (Ledergerber & Dessimoz, 2011; Pfeiffer et al., 2018; Sleep, Schreiber, & Baumann, 2013), missed nucleotides in *low‐complexity regions* (H. Li, 2014), index hopping (“bleeding”) (Vodák et al., 2018), and DNA damage during library preparation (Chen, Liu, Evans, & Ettwiller, 2017) caused by, for example, 8‐oxo‐G formation when using acoustic shearing or oxidative stress during probe hybridization (Newman et al., 2016; Park et al., 2017) and decreased coverage in regions of very *high or low CG content* (Sleep et al., 2013). Li (2014) showed that a large fraction of systematic errors found in variant callsets were not due to sequencing errors, but erroneous realignments in low‐complexity and repetitive regions (about 2% and 45% of the human genome, respectively), as well as the incompleteness of the reference genome with respect to the analyzed sample. While repetitive regions lead to ambiguous alignments of short reads and thus increase the likelihood of assigning a read to the wrong locus, low‐complexity regions also cause misalignment of reads at the correct position (Cordaux & Batzer, 2009; H. Li, 2014). Furthermore, many aligners tend to misalign indels close to the end of a read, as their scoring function favors mismatches over gap openings.

### 1.1 Strategies for identification of systematic genotyping errors

A multitude of variant calling algorithms that apply various strategies to reduce the false positive rate (FPR) have been developed. Commonly used tools for germline variant prediction include GATK HaplotypeCaller (McKenna, 2009), Samtools mpileup (H. Li, 2011), Freebayes (Garrison & Marth, 2012), or Varscan (Koboldt DC, Larson DE, 2013; Koboldt et al., 2009). Other tools, for example, Strelka (Saunders et al., 2012), VarScan2, and MuTect (Cibulskis et al., 2013), specialize in somatic mutation calling using tumor‐normal pairs. Most of these methods apply Bayesian statistics (e.g., Bayesian classifiers) to compute genotype likelihoods (Garrison & Marth, 2012; Van der Auwera et al., 2013), or, in case of somatic mutations, the likelihood of the variant model (Cibulskis et al., 2013). Some systematic alignment issues can be addressed by haplotype‐based variant calling as performed by FreeBayes (Garrison & Marth, 2012). Issues with gapped alignments around indel alleles are tackled by alignment post‐processing, using either multiple‐read realignment or local assembly (DePristo et al., 2011; Van der Auwera et al., 2013). Still, stringent post‐filtration of callsets using machine learning‐based error models (e.g., Variant Quality Score Recalibration [VQSR] (Carson et al., 2014; Van der Auwera et al., 2013)) or thresholds on various call quality statistics (e.g., genotype quality, read depth, variant allele frequency (VAF) (Li, 2014)), clustered variants, Fisher strand bias (Guo et al., 2012) is a necessity. Other strategies include removal of variants in low‐complexity regions as well as in repeats incompletely represented in the reference genome (typically indicated by significantly increased read coverage) (Carson et al., 2014; Li, 2014). However, a general issue of many post‐filtration strategies is their use of hard thresholds for the various quality metrics, where small changes can dramatically influence false negative and false positive rates, or their dependence on large sample sets to be effective (e.g., VQSR) (De Summa et al., 2017; Lek et al., 2016).

Here, we present a new strategy to identify systematic sequencing or alignment errors leading to false variant calls, which is based on the recurrent and significant deviation of observed to expected allele balance (AB) in a genomic position across large control cohorts. This signature, termed allele balance bias (ABB), was found in 0.03% of all exonic positions, 4% of high confidence germline SNV calls in 987 exomes, and 8% of somatic SNV calls in 200 tumor‐normal pairs. We present two algorithms: one for computing ABB for all positions of the exome (or genome) using large cohorts of WES (or WGS) samples, and one for refining candidate gene lists generated by RVAS. We have trained an ABB model for the human exome optimized for the use in clinical exome diagnostics and rare variant association testing in coding genes. Finally, we provide ABB genotype callability scores for all positions of the human exome.

## 2 MATERIAL AND METHODS

### 2.1 Whole‐exome sequencing and data analysis

We have analyzed 1,197 germline samples assembled from various genetic disease and cancer studies, including case and control cohorts sequenced at the CRG‐CNAG, Barcelona. Included individuals are of European ancestry. Exome capture has been performed using five different in‐solution capture methods: Agilent SureSelect versions 35 MB, 50 MB, 71 MB, and V5 and Roche‐Nimblegen SeqEz v3 (detailed information on samples and library preparation can be found in Supporting Materials and Supporting Information Table S1). We removed regions showing less than an average of 10× read coverage across samples analyzed using the same kit. For variant analysis, we extended captured regions by 50 bp upstream and downstream flanking regions. Sequencing was performed on Illumina HiSeq2000 or HiSeq2500 using 2 × 100 bp paired end reads (Bentley et al., 2008). Reads were aligned against the human reference genome (hg19) using BWA‐MEM (Li, 2013; Li & Durbin, 2009). Alignment post‐processing was performed according to GATK best practice guidelines (Van der Auwera et al., 2013), including PCR duplicate marking, Indel realignment, and base quality recalibration (Bao et al., 2014). Variant calling was performed using GATK HaplotypeCaller v3.3(McKenna, 2009; Van der Auwera et al., 2013). Variants with genotype quality below 20 or Fisher strand bias (FS) in the top 10 percentile were removed. For benchmarking purposes, we generated two callsets, one with and one without applying GATK VQSR filter (tranche threshold of 99.9%). See *Accesion Numbers* section for available data.

### 2.2 Deviation of observed from expected AB

We investigated the relationship between recurrent deviation of observed from expected AB, systematic errors and FP SNV calls in whole‐exome sequencing (WES) data. AB describes the fraction of reads supporting the alternative allele in a focal position (AB = alternative read count/total read count at focal position). When sequencing diploid species, heterozygous genotypes are expected to show an AB close to ∼0.5. We modeled read distribution for heterozygous genotypes using a binomial distribution *Binomial*(D, ∼0.5) (Guo et al., 2013; Nothnagel et al., 2011; O'Fallon, Wooderchak‐Donahue, & Crockett, 2013). Homozygous genotypes are expected to have close to 100% of reads supporting the same allele, with the amount of deviating reads depending on the sequencing and alignment error rate and other variables. We modeled the expected read distribution for homozygous reference using zero inflated beta distribution and for homozygous alternative using one inflated beta distribution, where AB would be inside the range [0, 1] (Ospina & Ferrari, 2012). The corresponding probability density function is given by

beinf y;α,γ,μ,ϕ=α1−γ, if y=0,αγ, if y=1,(1−α)f(y;μ,ϕ), if y∈(0,1),

where *f* (*y*; μ, *φ*) is the beta density function, and *μ* and *φ* are the parameters that define the shape of the beta distribution. Note that, if *y* ∼ BEINF(*α*,*γ*,*μ*,*φ*), then *P*(*y *= 0) = *α*(1 − *γ*) and *P*(*y *= 1) = *αγ* (Ospina & Ferrari, 2009), where *α* is the mixture parameter and *γ* represents the parameter of the cumulative distribution function of a Bernoulli random variable. Here, *y* represents the AB variable. Parameters for expected AB distributions for each diploid genotype class have been estimated using post‐VQSR variant calls from GATK *HaplotypeCaller* by maximum (penalized) likelihood estimation (*GAMLSS* R package). We genotyped every position of the exomes of 1,197 samples by comparing observed to expected AB, obtaining one *P* value for each of the three possible diploid genotypes. We assumed that the greatest *P* value represents the most likely genotype of a focal sample and position. Given this genotype, we measured the deviation of observed from expected AB (devAB equal to AB − 0 for homozygous reference, |AB − 0.5| for heterozygous and |AB – 1| for homozygous alternative).

### 2.3 Allele balance bias

Using devAB at a focal position in hundreds of samples, we can identify positions showing recurrent deviation of observed from expected AB, termed ABB. To quantify and model ABB, we processed a training cohort of 987 germline WES samples, leaving 200 germline samples for validation and testing of the model (randomly chosen from normal tissue exomes of 450 Chronic Lymphocytic Leukemia [CLL] patients) (Puente et al., 2015). For Sanger validation, we used 10 independent samples, which were all obtained after ABB training (and for which ample amounts of DNA were available) (see Supporting Material and Supporting Information Tables S1 and S2 for detailed sample information). Note that the influence of somatic mutations on model training can be neglected, as training is performed only on healthy blood or normal tissue samples, in which somatic mutations are expected to be extremely rare and not recurrent across samples.

We obtained pileup files (samtools mpileup version 1.1) for each sample and collected read depth and allele counts for more than 80 Million exonic positions. We computed alternative allele fractions, most likely genotype and devAB for positions covered by at least 20 reads with base quality ≥20 (considered informative). Positions with less than 80 informative samples were excluded from further analysis. We calculated three measures of ABB strength for each position of the exome based on sample‐wise devAB, termed *RdAB1*, *RdAB2*, and *RdAB3. RdAB1* represents the mean of *devAB* across all informative samples at a focal position. *RdAB2* measures the fraction of samples with a significant deviation from the expected distribution of the most likely genotype. *RdAB3* represents the arithmetic mean of −log_10_ (*P* value) across all samples at a focal position.

### 2.4 ABB‐based genotype callability model

To integrate the three measures of ABB into one genotype callability score, we trained a logistic regression model using the variant calls of 200 samples not used for defining *RdAB1*, *RdAB2*, and *RdAB3*. This variant callset was obtained using GATK HaplotypeCaller as described above but omitting VQSR to allow the capture of an increased number of potentially false calls for training purposes. We focused our analysis on heterozygous variants with at least 60 affected samples, resulting in 27,953 positions. To obtain the labels, we calculated the mean devAB values for all 27,953 positions, and split them into two sets using Gaussian Mixture Modeling (*mclust* R package, R version 3.2.3): non‐recurrently deviated AB positions (labeled 0), and recurrently deviated AB positions (labeled 1). Two thirds of the data points (18,635 positions) were used for ABB model training (training set) 4,659 positions were used for validation of the resulting ABB model and 4,659 positions for final evaluation of the LR1 ABB model (test set). The logistic regression model LR1 uses *RdAB1*, *RdAB2*, and *RdAB3* as features to predict the labels obtained by Gaussian Mixture Modeling. It returns the probability of a variant site belonging to the label 1 (recurrently deviated AB positions) using the R function *glm* with family = “binomial”:

$$\begin{array}{ccc} \log\left(y/\left(1-y\right)\right) & = & \beta_{0}+\beta_{1}\cdot rdAB1+\beta_{2}\cdot rdAB2+\beta_{3}\cdot rdAB3\\ & & +\beta_{4}\cdot rdAB2\cdot rdAB3 \end{array}$$

with *y* = 1 (recurrently deviated positions).

Subsequent to the estimation of the logistic regression parameters *β_i_*, we calculated F1 scores at different probability levels and chose the maximum as optimal cutoff to assign labels. For this cutoff, we calculated precision, recall, *F* score, and FPR (see formulas in Supporting Material), as well as Precision‐Recall Area Under the Curve (PR‐AUC) and ROC Area Under the Curve (ROC‐AUC) values.

**Figure 1:**
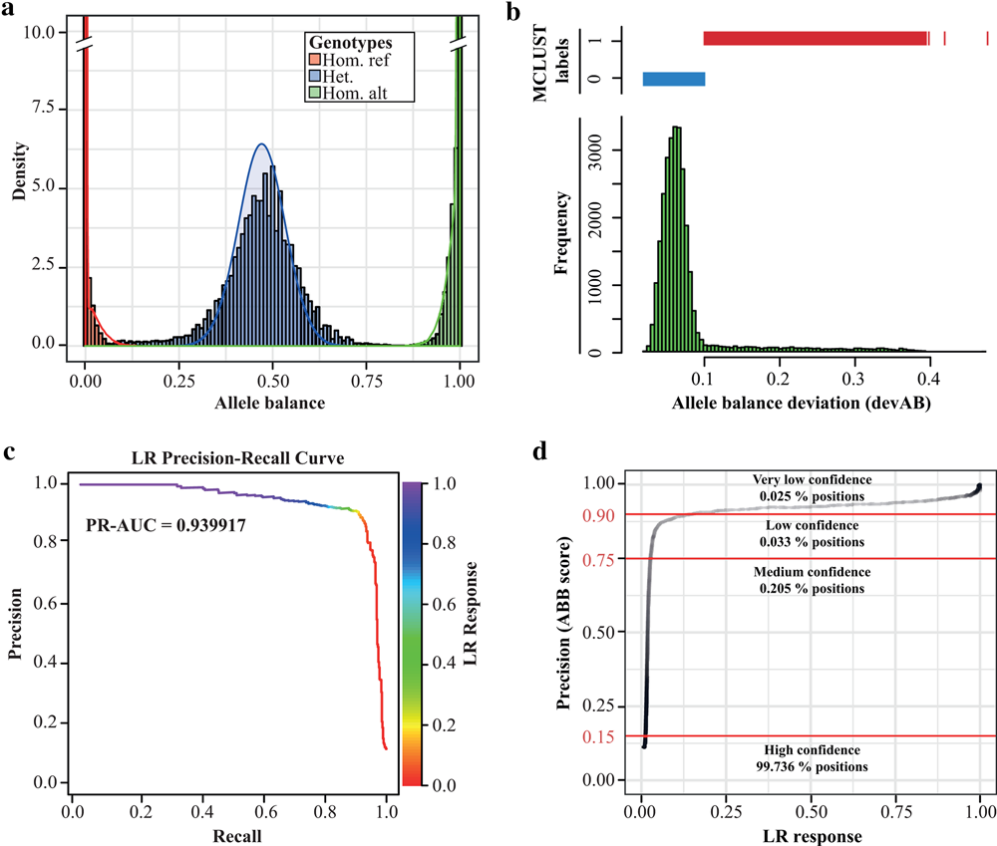
(a) Observed (bars) and expected (density) allele balance (AB) distributions split by genotype. (b) Gaussian mixture model of the allele balance deviation devAB, separating non‐deviated (0) and deviated (1) positions. (c) Precision‐Recall curves and PR‐AUC for the linear regression model LR‐1. The color gradient on the right shows the LR response value (probability to belong to class 1) obtained by logistic regression. (d) Correlation of LR response and precision. Precision was measured in the test and validation sets using labels defined by the GMM. Confidence levels were defined by visual inspection

Using LR1, we calculated the probabilities to belong to the label *recurrently deviated AB* for each position of the human exome. We mapped the LR response value (probability) to precision values using the results obtained for validation and test sets. The resulting score, termed ABB genotype callability score, can be applied to estimate the callability of any position of the exome, with higher values indicating a higher likelihood of systematic errors. Based on visual inspection of the LR response to precision curve (Figure 1d) we defined four genotype callability levels, comprising high confidence (ABB <= 0.15), medium confidence (0.15 < ABB <= 0.75), low confidence (0.75 < ABB <= 0.9) and very low confidence (ABB > 0.9) positions.

### 2.5 Evaluation of ABB by Sanger sequencing

To benchmark the ability of ABB to identify FP variant calls, we randomly selected and Sanger sequenced 209 “suspicious” SNP calls (0.2 <= AB <= 0.35) from 10 samples not used for model training, selection or evaluation (see Supporting Materials and Supporting Information Table S3 for more details). SNPs were sampled to similarly represent all four ABB genotype callability levels (42 high confidence, 73 medium confidence, 46 low confidence, and 48 very low confidence SNPs). Additionally, 45 Sanger validations of novel disease variant candidates obtained within previous studies were included in the benchmarking. We compared FP and failure rates (failed Sanger sequencing or ambiguous base call) between ABB bins and computed a ROC curve. Note that all variants selected for Sanger validation passed the GATK VQSR, fisher strand, and minimum AB filters following the GATK best practice guidelines.

### 2.6 Relationship of ABB with other genomic features, quality measures, and variant databases

Using variant calls generated by GATK HaplotypeCaller for 10 samples (filtered with VQSR), we interrogated the correlation of ABB, fisher strand bias and transition–transversion ratio (Ti–Tv) for sites likely affected by systematic errors (ABB >= 0.9) using Wilcox test and Pearson's chi‐square test, respectively. Using chi square Pearson's test, we further investigated the enrichment of very low confidence variants in different public databases (dbSNP version 146, ExAC version 0.3v, 1000GP phase 3, EVS ESP6500), compared with the fraction across all informative positions of the exome. Similarly, we compared the relation of ABB with simple sequence repeats (SSRs) and tandem duplications. For that, we randomly selected a set of positions of very low (ABB >= 0.9) and high (ABB < 0.15) confidence and compared the fraction of SSRs and tandem duplications using a Chi‐square Pearson's test. We visualized the intersection of positions labeled un‐callable by Genome in a Bottle (GIAB v3.3.2 High‐Confidence regions (Zook et al., 2016)) and ABB using Venn diagrams and compared the performance in filtering FP SNP calls on 209 sites validated by Sanger sequencing.

We interrogated the enrichment of very low confidence sites in somatic variant calls using tumor‐normal paired data from 200 CLL patients, whose normal sample had not been used for ABB model building (germline variant positions used in model building were also excluded from enrichment analysis). Somatic SNVs were predicted using MuTect (Cibulskis et al., 2013). We measured the enrichment of high, medium, low and very low confidence sites in somatic mutation calls compared to their exome‐wide expectation and the enrichment of each quality bin in Cosmic and dbSNP using in both cases Chi‐Square Pearson's test.

### 2.7 Quality control for RVAS analysis

To test if ABB can identify false associations from RVAS, we developed Association‐ABB, a method that tests if ABB can explain the difference in alternative allele counts (“burden”) for a gene between cases and controls, and hence the genotype–phenotype association hypothesis can be rejected. In summary, the algorithm computes gene‐wise aggregated measures of ABB in case and control cohorts in order to detect false associations arising from an uneven impact of ABB on variant calls in cases compared with controls. The algorithm takes as input the variants from the candidate genes generated by RVAS, and, for each variable position, identifies cases and control samples for which the variant caller might have missed or falsely predicted the alternative allele. Possibly “missed” alternative alleles are defined as homozygous reference calls for which the *P* value within the homozygous AB zero‐inflated beta distribution is less than 0.05. First, for each variant in RVAS candidate genes, we test if the ratio of “called” compared with “missed” alternative genotypes is biased between cases and controls (Fisher exact test). Second, for each RVAS candidate gene, three tests are performed: (1) called‐missed ratio test (similar to the variant‐wise test but aggregating all rare variants per gene); (2) rerunning the association test but including the “missed” calls as variants (Chi‐Square Pearson's test aggregating all variants per gene); and (3) rerunning the association test but removing significantly AB biased sites (Chi‐Square Pearson's test). Genes with FDR lower than 0.1 in the called‐missed ratio test or genes not significantly associated (FDR > 0.1) when adding “missed” variants or removing ABB variants are considered potential FP associations. Association‐ABB is available as part of the ABB package at https://github.com/Francesc-Muyas/ABB. We have tested Association‐ABB on an RVAS study for CLL (see Supporting Material for details) in which the comparison of germline variants from 437 cases and 780 controls by SKAT‐O (S. Lee, Wu, & Lin, 2012), Burden (B. Li & Leal, 2008; Madsen & Browning, 2009; Price et al., 2010), MiST (Sun, Zheng, & Hsu, 2013), and KBAC (Liu & Leal, 2010) association tests resulted in 43 CLL associated candidate genes, 10 of which were labeled as FP by Association‐ABB.

## 3 RESULTS

We have developed a genotype callability score for NGS analysis based on the recurrent deviation of observed from expected AB, termed ABB. Using an ABB model trained on 987 WES datasets, we precomputed ABB genotype callability scores for more than 81 Million positions of the human exome. We did not observe biases in genotype callability rates between kits when focusing on regions well‐covered in all kits (average coverage ≥ 10, see Supporting Material and PCA in Supporting Information Figure S1). To evaluate the performance of ABB on identification of systematic errors and FP genotype calls, we used an independent set of 210 WES cases and Sanger validation. In addition, we demonstrate that ABB correlates with various measures of sequencing and alignment errors and show that public variant databases are enriched for systematic genotyping errors.

### 3.1 Training and evaluation of the ABB model

We hypothesized that systematic sequencing or alignment errors lead to recurrent deviation of ABs in affected genomic positions across hundreds of samples. To test this hypothesis, we trained, evaluated, and tested a logistic regression model distinguishing positions with and without recurrently and significantly deviated AB, which integrate three measures of AB deviation (see section *Materials and Methods*; Figure 1a–c). All coefficients of the logistic regression significantly contributed to the selected model (*P* values ≪ 0.01). We calculated F1 scores at different probability levels and chose the maximum (LR response of 0.13 at F1 of 0.91) as cutoff to assign labels. On the evaluation set of 4,659 variants, LR1 showed a precision of 0.893, recall of 0.915, PR‐AUC of 0.940, ROC‐AUC of 0.980, and F1 of 0.904 for the optimal cutoff (Figure 1c and Supporting Information Table S4). An independent test using the remaining 4,659 positions not used in any previous step showed similar performance (precision of 0.898, recall of 0.899, PR‐AUC of 0.933, ROC AUC of 0.975, F1 of 0.899), demonstrating that the model was not over‐fitted and can be generalized to novel datasets. Based on the correlation of LR response values and precision (Figure 1d), probabilities obtained for each position of the exome were transformed to the precision of predicting systematic errors, which we finally use as ABB score. Higher ABB score values indicate a higher probability to obtain systematic errors in variant calling, with ABB > 0.75 considered low confidence positions (0.033% of the exome) and ABB > 0.9 considered very low confidence positions (0.025% of the exome).

### 3.2 ABB genotype callability filter for germline and somatic variant calling

**Figure 2:**
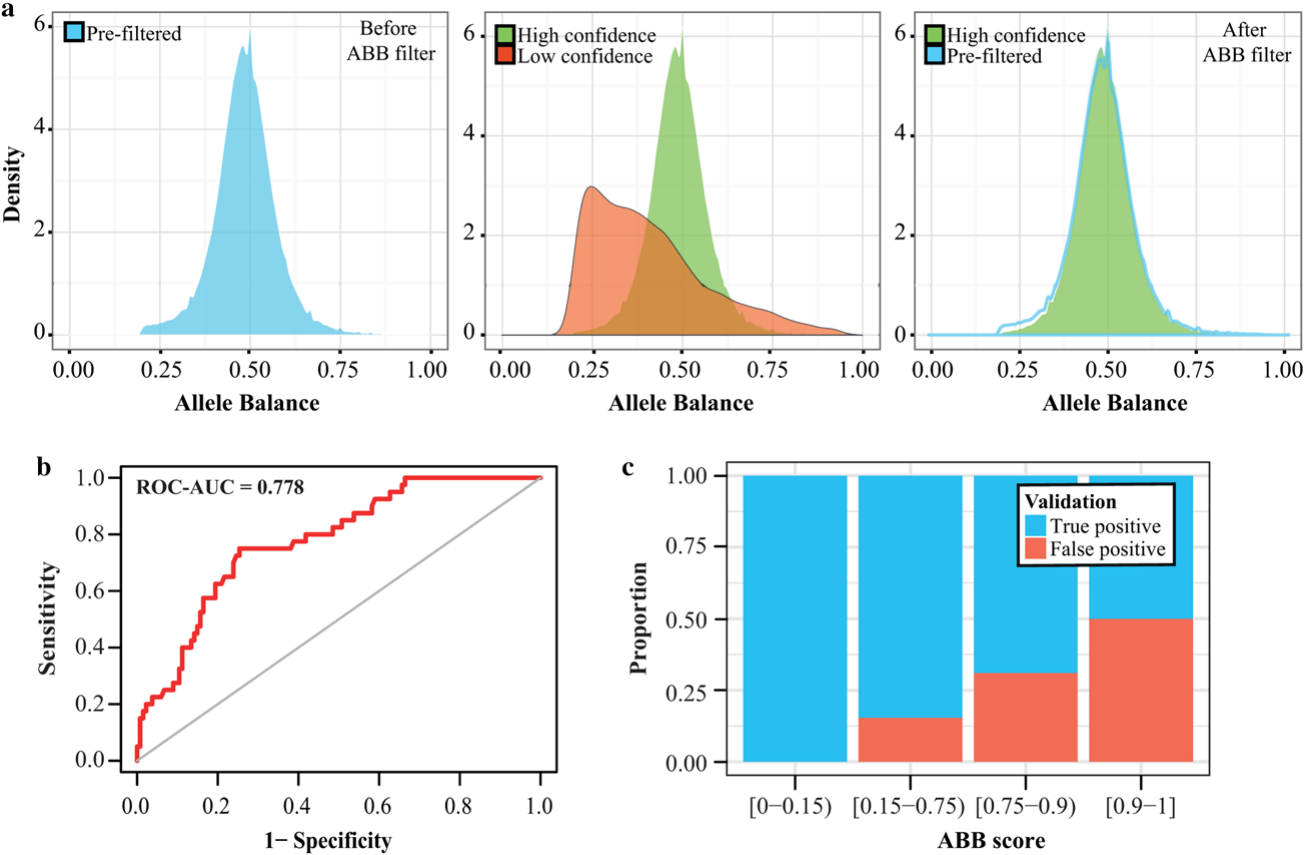
(a) ABB classifications of heterozygous SNPs reported by GATK HaplotypeCaller. Shape of AB distribution of variants identified by GATK + VQSR (left); AB distribution of low (red) compared with high (green) confidence positions (middle); and AB distribution after ABB filtering (right). (b) ROC curve of Sanger validation results compared with ABB (AUC = 0.778). (c) Proportion of True Positive (TP) and False Positive (FP) variants in four ABB genotype callability ranges

To evaluate if the use of ABB as genotype callability filter leads to improved variant callsets, we applied an ABB very low confidence filter (ABB > 0.9) to variants predicted by GATK HaplotypeCaller with VQSR filtering. Using a callset for 10 samples not used during ABB model training, evaluation, or testing, we found that 13,168 out of 346,894 (3.80%) variant sites overlapped with ABB very low confidence sites (compared with 0.025% of all exonic positions, *P* value < 10^−16^; Table 1 and Supporting Information Figure S2), with an average of 1,317 (3.80%) variants per sample. Surprisingly, 44.59% of known germline variants were flagged as medium confidence sites. We found that polymorphisms with ABB medium confidence are enriched for high population AF in 1000GP (mean of 26%), while polymorphic sites with ABB high confidence are mostly rare variants (population AF of 0.08%, Wilcox test *P* value < 10^−16^), reflecting that heterozygous sites are generally harder to call than homozygous sites due to a larger standard deviation of the heterozygous VAF distribution. The distribution of AB across all 346,894 positions showed a “belly” on the left of the normal distribution (AF between 0.2 and 0.35; Figure 2a‐left). Specifically, the VAF of SNVs classified as very low confidence was skewed (Figure 2a‐middle, red distribution), with a large fraction showing VAF between 0.2 and 0.35. Application of the ABB filter resulted in a “clean” normal distribution (Figure 2a‐right).

**Table 1:** Distribution of ABB genotype callability levels in the whole exome, germline SNV calls, and somatic SNV calls

ABB callability	Whole exome	Germline SNV	Somatic SNV
High confidence [0–0.15)	99.736%	44.955%	80.286%
Medium confidence [0.15–0.75)	0.205%	44.585%	5.771%
Low confidence [0.75–0.9)	0.033%	6.665%	5.865%
Very low confidence [0.9–1]	0.025%	3.796%	8.077%

The transition–transversion ratio expected to be around 3 in exomes was significantly smaller for very low (ABB > 0.9) compared with high (ABB < 0.15) confidence positions (1.76 compared with 2.54, *P* value < 10^−16^). Moreover, low confidence sites showed significantly increased fisher strand bias (*P* value < 10^−16^). Furthermore, very low confidence sites were enriched for segmental duplications (27.10% of positions, *P* value < 10^−6^) and SSRs (16.27% of positions, *P* value < 10^−6^), compared with high confidence sites (2.50% and 0.95% of positions for segmental duplications and SRRs, respectively) (Supporting Information Table S5).

In order to test the applicability of ABB for improving somatic mutation callsets, we generated somatic SNV calls for 200 CLL tumor‐normal pairs using MuTect and obtained precomputed ABB scores for each site (importantly, note that the ABB model is not calculated using tumor tissues, but scores are obtained from the germline‐based model described above). ABB low and very low confidence positions represented 5.9% and 8.1% of the somatic mutation calls, respectively (Table 1), representing a significant increase of very low confidence positions compared to 3.8% observed for germline variant calling (*P* value < 10^−16^) and the exome‐wide expectation (*P* value < 10^−16^). Interestingly, 45.38% of the very low confidence mutations were found in dbSNP. This proportion was significantly higher (*P* value < 10^−16^) than the fraction of high confidence somatic mutations in dbSNP (9.51%; Table 2), pointing at a systematic introduction of errors in dbSNP.

**Table 2:** Enrichment of somatic SNV calls in dbSNP and Cosmic, separated by ABB callability range

ABB callability	Novel	Cosmic	DbSNP
All SNVs [0–1]	80.89%	4.53%	14.58%
High confidence [0–0.15)	85.60%	4.89%	9.51%
Mid confidence [0.15–0.75)	68.08%	3.47%	28.45%***
Low confidence [0.75–0.9)	67.95%	4.06%	27.99%***
Very low confidence [0.9–1]	52.60%	2.02%*	45.38%***

Row 1 shows results for the complete call set used as baseline.

*P value < 10E−3.

***P value < 2E−16.

To demonstrate that our model is not falsely labeling real somatic mutations as systematic errors we intersected positions marked as systematic errors (ABB >= 0.9) with 1896 somatic mutations validated in two studies (Papaemmanuil et al., 2014; Tarpey et al., 2013) and 341 well‐known cancer driver mutation hotspots (Chang et al., 2016). We found a minimal overlap of two out of 1,896 and zero out of 341, respectively. There was no significant difference between the fraction of systematic errors identified in the whole exome (Table 1) and the set of validated somatic SNV positions (*P* value = 0.1421), demonstrating that ABB does not misclassify true somatic mutations as systematic errors more than expected by chance (Supporting Information Table S6).

### 3.3 Sanger sequencing‐based evaluation of ABB scores

We next evaluated if ABB scores correlate with the probability of calling FP variants. To this end, we validated by Sanger a set of randomly selected 209 heterozygous SNPs predicted by GATK HaplotypeCaller with VQSR in 10 samples, which had AB between 0.2 and 0.35, and that were sampled equally from each of the four ABB genotype callability levels (see section *Materials and Methods*). We found that ABB genotype callability levels correlated with FPR (Figure 2c, Table 3, and Supporting Information Table S7). Furthermore, ABB scores were predictive of FP calls (ROC‐AUC = 0.778; Figure 2b). Although the original variant callset produced by GATK HaplotypeCaller and VQRS could be considered high quality (tranche threshold of 99.9%), we found an FPR of 50% in the very low confidence set and 31% FPR in the low confidence set, while high and medium confidence positions showed only 0% and 15.4% FPR, respectively. Interestingly, the fraction of failed (ambiguous) Sanger sequencing experiments was significantly higher for the low confidence range when compared against high confidence range (*P* value < 0.025; Table 3), indicating that low‐complexity regions and repeats constitute one of the underlying issues, as these also affect efficiency of Sanger sequencing (Kieleczawa, 2006).

**Table 3:** Results of Sanger validation grouped by ABB genotype callability levels

ABB callability	SNVs	TP	TP rate	FP	FP rate	Failed	Fail rate
High confidence [0–0.15)	42	38	100.00%	0	0.00%	4	9.52%
Mid confidence [0.15–0.75)	73	55	84.62%	10	15.38%	8	10.96%
Low confidence [0.75–0.9)	46	20	68.97%	9	31.03%	17	36.96%
Very low confidence [0.9–1]	48	21	50.00%	21	50.00%	6	12.50%

Failed Sanger sequencing experiments were ignored for the FP and TP rate calculation.

Next, we compared the performance of ABB and the GIAB callability classifier on identification of FP calls. Considering all exons of the autosomes (79,660,917 bp included in the ABB model), GIAB classifies 75,442,680 sites as callable, leaving 4,218,237 sites as “un‐callable.” In comparison, ABB classifies 46,396 sites as low or very low confidence (ABB >= 0.75, considered un‐callable from here on). Of the 46,396 sites classified un‐callable by ABB, 52% are classified as callable by GIAB, demonstrating that the two methods are not redundant (Supporting Information Figure S3). Of the 40 GATK SNV calls confirmed as false by Sanger sequencing (out of the 209 sites evaluated by Sanger) ABB identified 30 (75%), while GIAB identified 23 (57.5%), although ABB filters substantially fewer sites across the whole exome than GIAB (40 kb vs. 4 MB) (see Supporting Materials and Supporting Information Table S8 for details). In a similar manor, we showed that both GQ and Hardy‐Weinberg Equilibrium provide complementary, but not redundant sources of information for filtering of variant calls (details in Supporting Materials, Supporting Information Figures S4–S6, and Supporting Information Tables S9 and S10).

Independent from the random Sanger evaluation, we obtained validation data for disease variant candidates (*novel* mutations) prioritized in in‐house analysis of various disorders (data unpublished). In each study, almost 50% of candidate variants were found to be FPs by Sanger validation. ABB labeled 11 out of 17 (64.7%) FP calls as low or very low confidence sites, and 6 FPs as medium confidence, while all TP variants fell into the high confidence category (Supporting Information Table S11). We observed a large margin between ABB for TPs (average of 0.115) and FPs (average of 0.666).

### 3.4 ABB scores of variants in public databases

Public variant repositories differ in the way included variants are called, quality controlled, and selected for integration. For instance, the 1000GP, ExAC/GnomAD, and EVS databases are created in a consistent manner, using a defined pipeline for all samples (Lek et al., 2016). However, dbSNP does not dictate any specific variant prediction method or quality control procedure and contains both germline and somatic variants. Hence, we hypothesized that although all variant databases may contain systematic errors, dbSNP is specifically affected by FPs due to its inconsistent quality parameters, as previously suggested (Musumeci et al., 2011). We found that very low confidence positions were significantly enriched in several public variant databases (all *P* values < 10^−16^; see Table 4). As expected, we found the strongest enrichment of systematic errors (ABB > 0.9) in dbSNP (15.9 times more than expected). As many variant analysis pipelines use the same tools as employed for generating 1000GP, ExAC, GnomAD, or EVS, one should be cautious when considering variants found in these databases as validation gold standard for variants in newly generated callsets. As we expect to see systematic errors repeatedly, this circularity issue (validation using false variants predicted by the same tools) can lead to a “self‐fulfilling prophecy,” where false variants are established as true positives in public databases and potentially influence disease studies in the future.

**Table 4:** Enrichment of ABB very low confidence (VLC) positions in public variant databases

Database	Total positions	VLC Obs.	VLC Freq. Obs.	Ratio Obs./Exp.
Exome	81,609,944	20,725	0.03%	1
dbSNP	3,172,724	12,787	0.40%	15.87*
EVS	1,840,709	1,114	0.06%	2.38*
1000GP	2,653,982	4,690	0.18%	6.96*
EXAC	2,662,396	3,510	0.13%	5.19*

The fraction of VLC positions in the exome was used as expected value.

*P value < 10E−16.

### 3.5 Filtering candidate genes from RVAS

WES is frequently applied to identify causal variants for genetic diseases, using rare variant association tests in large cohorts or analysis of affected families and parent‐child trios. Although ABB can be used generically to filter results of variant callsets, we have in addition developed a custom algorithm, Association‐ABB, for identification of cohort‐specific false associations caused by systematic errors (see section *Materials and Methods*). We hypothesized that false associations can be introduced in case–control studies due to (1) a bias in systematic errors between cases and controls, leading to an uneven burden of false variant calls, or (2) copy number variants enriched in cases or controls, for example, due to biased population structure. Therefore, we reanalyzed variants in candidate genes in order to identify associations better explained by biases in the burden of systematic errors.

The Association‐ABB evaluation was performed on candidate genes resulting from an RVAS for CLL using WES of 437 CLL normal samples from ICGC‐CLL (Quesada et al., 2011) and 780 control samples. In the 43 resulting candidate genes identified by SKAT‐O and Burden (see section *Materials and Methods*), Association‐ABB labeled 24 out of 739 SNPs as affected by a bias in systematic errors that were called as variants to a different extent in cases and controls (“called‐missed ratio fisher test,” see section *Materials and Methods* and Supporting Information Table S12). In addition, these variants showed a high ABB score (average of 0.8670). We next performed a gene‐wise aggregated test of biased sites and found that 10 out of 43 candidate genes were likely false associations (Supporting Information Table S13). In brief, we tested if the RVAS association was still significant when (1) biased sites were excluded or (2) potentially missed calls were added to the test (see section *Materials and Methods*).

One example gene, *CTDSP2*, is shown in Supporting Information Figure S7. We observed that a different AB distribution in cases and controls in 8 biased positions led to an imbalanced genotyping efficiency of GATK HaplotypeCaller, explaining the significant association of this gene with CLL in the RVAS test. Comparing called versus potentially missed SNVs, we found a significant enrichment of missed calls in the controls, that is, positions called as homozygous reference, although more than expected reads showed the alternative allele. However, not only cases showed an enrichment of calls with significantly deviated AB in heterozygotes (AB around 0.25), but also controls that had been enriched using Agilent SureSelect, while controls prepared with NimblegenSeqEz were “clean” (Supporting Information Figure S8 and Supporting Material). We conclude that a systematic issue with few target regions of one enrichment kit introduced the false RVAS call.

The AB patterns between cases and controls in the gene CDC27 look similar to CTDSP2, as shown in Supporting Information Figure S9, although this enrichment was not associated to the capture method as CTDSP2 (see Supporting Information Figure S10). Moreover, literature search revealed that this gene frequently harbors FP SNVs (Jia et al., 2012), likely caused by multiple novel retroduplications (Abyzov et al., 2013). Indeed, we found that cases with deviating AB also showed significantly increased coverage on the exons affected potentially by retroduplications (Supporting Information Figure S11) (see Supporting Material for detailed explanation of this section).

Association testing when removing problematic site in CDC27 or CTDSP2 (“cleaning”) or when adding potentially missed calls led to non‐significant association tests. In summary, ABB identified retrotransposition as well as exome hybridization kit‐related systematic errors causing false associations in an RVAS study of CLL.

## 4 DISCUSSION

In this work, we present a new genotype callability filter for exome or genome sequencing analysis, which is based on the recurrent and significant deviation of observed from expected AB at a genomic position across hundreds of NGS datasets. We termed the underlying phenomenon ABB. Up to 4% of the positions called as germline variants and 8% of positions called as somatic mutations by state‐of‐the art methods show ABB scores indicative of systematic errors. We used Sanger validation of random germline calls and of disease variant candidates to show that ABB correlates with the likelihood to identify FP SNVs, with more than 50% FPR in the lowest genotype confidence range. Furthermore, ABB low and very low confidence positions show a low transition–transversion ratio (TiTv) (Freudenberg‐hua et al., 2003; Pattnaik, Vaidyanathan, Pooja, Deepak, & Panda, 2012) and are highly enriched for low‐complexity regions, supporting the hypothesis that LCRs are responsible for a large fraction of systematic errors (H. Li, 2014). Nonetheless, our findings indicate that several other issues can cause systematic errors, including incomplete reference genomes and unknown CNVs or segmental duplications, among others.

Although the accuracy of variant callers has been optimized since the introduction of NGS, there are still systematic errors that cannot be identified by the current set of QC parameters. While ABB shows partial correlation with other QC measures like Fisher strand bias and LCRs, none of these parameters can identify the complete set of positions flagged by ABB, making ABB a valuable addition to the QC filter setup. Interestingly, we found that sites prone to systematic errors are highly enriched in public variant databases. As these databases are often used for benchmarking purposes this can lead to a “fixation” of false calls, and can skew benchmark results. dbSNP by far showed the highest enrichment of systematic errors, as suggested previously (Musumeci et al., 2011), demonstrating that variant callsets created consistently by a defined and reproducible pipeline and parameter setting (e.g., 1000GP, ExAC/GnomAD, EVS) are preferable. Systematic errors constitute an even bigger issue for somatic SNV calling. Even if ultra‐deep sequencing is used to identify sub‐clonal mutations, systematic errors, other than random errors, will still lead to false somatic SNV calls (Griffith et al., 2015). Indeed, we observed that close to 14% of somatic SNVs called by MuTect were classified as ABB low or very low confidence sites, a significantly larger fraction than observed for germline variant calling or expected on an exome‐wide level. Moreover, these FP mutations are again highly enriched in dbSNP. Considering the importance of predicted point mutations for cancer diagnostics and optimal treatment selection, removal of these FP calls is essential for the applicability of NGS in precision oncology.

Systematic FP calls can lead to false associations of genes with disease. Using Sanger validation of disease gene candidates prioritized in previous projects, we demonstrated that high‐confidence ABB sites are 100% true positives, allowing to reduce the cost of Sanger validation by omitting validation of these sites. At the same time, ABB identifies up to 65% of false candidates (considering low or very low confidence sites, up to 100% if also considering medium confidence as FP). We further demonstrate how systematic errors resulting in false associations can be identified by Association‐ABB in a cohort specific manner. We found that in a rare variant association test for CLL around 25% of candidate gene associations were better explained by uneven burden of systematic errors in cases and controls. We further hypothesized that systematic SNV calling errors were introduced by an un‐annotated CNV in at least couple of candidate genes, indirectly pointing to the real cause of the genotype–phenotype association.

The current ABB model has been built using alignments generated by bwa‐mem. Hence, some systematic errors identified in our study might reflect specific alignment issues of bwa‐mem and might not be observed when using, for example, bowtie2. However, bwa‐mem is one of the most used aligners for human genomics, making our model directly applicable to a majority of projects using WES or whole‐genome sequencing of human samples. Nonetheless, one could retrain the ABB model for specific computational analysis pipelines, for other species, for whole genomes or using thousands of additional samples, a process we support by offering all scripts for generating dedicated ABB models (see *Availability* section).

In summary, our novel genotype callability estimator based on ABB can identify systematic variant calling errors not found by other measures and can improve the accuracy of germline and somatic variant sets as well as disease association studies in families or large cohorts.

## Supporting information

Supporting Material

Supp. Table S1. Sample information of the 1197 germline samples used in the ABB study.
Supp. Table S2. Sample information of the 200 tumor samples used in the analysis of the somatic variant calls.
Supp. Table S3. Primers design for Sanger sequencing run
Supp. Table S4. Model training, testing, and evaluation.
Supp. Table S5. Repetitive elements contingency (%) table in very low and high confidence sites.
Supp. Table S6. Overlap of validated somatic variants from three studies (Tarpey et al., 2013; Papaemmanuil et al., 2014; Chang et al., 2016) and known cancer driver mutations hotspots with positions labelled as systematic errors by ABB.
Supp. Table S7. Results of Sanger sequencing validation for 209 random sites.
Supp. Table S8. Classification of false positive SNV sites (as evaluated by Sanger sequencing) by GIAB and ABB. Sites are grouped by ABB confidence levels and GIAB classification. GIAB correctly classified 23 out of 40 and ABB 30 out of 40 false positives. 9/40 false positives are only found by ABB (shown in bold), 2/40 are only found by GIAB.
Supp. Table S9. Performance of ABB score and Hardy‐Weinberg equilibrium filter on Sanger‐identified false positive calls
Supp. Table S10. Performance of ABB score and Hardy‐Weinberg equilibrium filter on Sanger‐validated true positive calls
Supp. Table S11. Sanger validation of novel variants found in different cohorts of patients. Very low‐confidence mutations (ABB >= 0.9) are red colored and low confidence mutations (0.75 <= ABB < 0.9) are colored in orange.
Supp. Table S12. List of variant sites labelled as significant in the ABB association test based on Missed‐Called ratio (FDR) obtained from the missed‐called ratio test (methods).Table S12. List of variant sites labelled as significant in the ABB association test based on Missed‐Called ratio (FDR) obtained from the missed‐called ratio test (methods).
Supp. Table S13. Genes labelled as prone to false positive associations based on three test (methods): Missed‐Called ratio (FDR) obtained from the missed‐called ratio test; Association re‐genotyped (FDR) from association chi square test between cases and control including re‐genotyped variants; Association‐ABB (FDR) association analysis with chi square test removing prone to significant variant sites.
