# Supplementary material for: Allele balance bias identifies systematic genotyping errors and false disease associations
Source: Hum Mutat. 2018 Nov 23;40(1):115–26. doi: 10.1002/humu.23674 (PMC6587442; doi:10.1002/humu.23674)
Supplement: Supplementary file 1 — Supporting Material [file HUMU-40-115-s001.pdf]

Supporting Material

Formulas and nomenclatures

- TP ~ True positive
- FP ~ False positive
- FN ~ False negative
- TN ~ True negative
- Precision = $\frac{TP}{TP+FP}$
- Recall (sensitivity) = $\frac{TP}{TP+FN}$
- FDR (False discovery rate) = 1 – precision = $\frac{FP}{TP+FP}$
- F-score (F1) = $\frac{2TP}{2TP+FP+FN}$

Sample information

In total, 1197 WES samples were used in this study (see Supp. Table S1-S2 for sample details). 987 were used for ABB model training. For subsequent validation, we left 200 germline samples 'untouched' during training, that were randomly chosen from normal tissue exomes of 450 Chronic Lymphocytic Leukemia patients. We chose CLL cases for validation because the availability of WES data for tumor and normal tissue allowed us to validate the power of ABB as a quality filter for variant calls for both germline and somatic call sets. CLL tumor samples were obtained from fresh or cryopreserved mononuclear cells. To purify the fraction of cells affected by CLL (or MBL in a few cases, a precursor disease of CLL), samples were incubated with a cocktail of magnetically labeled antibodies directed against T cells, natural killer cells, monocytes and granulocytes (CD2, CD3, CD11b, CD14, CD15 and CD56), adjusted to the percentage of each contaminating population (Puente et al., 2015 for more details). Hence, purity of both tumor and normal samples is very high (>=99%).

For Sanger validation, we chose 10 new (germline) samples not included in training or validation before, and for which we had ample amounts of DNA available.

Reducing biases between capture kits

The main differences found between capture methods are the regions that are consistently well covered. Each kit version had specific sets of regions which are supposed to be covered by probes according to vendor specifications, but recurrently show no or low coverage. This issue led to consistent biases in genotype call rates between enrichment kits in these regions, resulting in a strong clustering of samples by kit version in SNP-based PCA analysis (data not shown). However, we observed that this bias almost completely disappeared when we focused only on regions that had at least 10x average coverage in any kit version (Supp. Fig. 1). In order to maximize the regions for which we could calculate ABB, we did not simply use the region intersection of all kits, but for each position of the exome determined all kit versions that show consistently good coverage (average depth >= 10x). Subsequently, all statistics required for ABB modeling at the focal position were obtained from the subset of kits reaching the minimum quality threshold. Hence, for some genomic regions the ABB model has been trained by less than 987 samples.

Variant prediction and Sanger validation

Germline variants used for validating the power of ABB as a quality filter were called using GATK-HC with VQSR and quality filtered using the parameters:

1. Genotype quality score with a threshold of >= 20 at individual sites
2. Read depth >= 10 at individual sites
3. Alternative allele frequency at individual sites: >= 0.2.
4. Call rate across the whole cohort: >=80%.
5. Average alternative allele frequency across all individuals showing the variant: >=0.25.
6. Phred-scaled p-value using Fisher's exact test to detect strand bias (10% worst removed)
7. Variant Quality Score Recalibration: VQRS tranche threshold of 99.9%

Primers for PCR amplification and sequencing for each variant were designed with *Primer3 (http://bioinfo.ut.ee/primer3-0.4.0/){1}* and confirmed for specificity with *Blat (https://genome.ucsc.edu/)*. Fragments were amplified by PCR followed by Sanger sequencing reaction on an *ABI3730xl* machine using *BigDye* (Applied Biosystems). Sanger sequences were analyzed using the CLC genomics workbench (*Qiagen*) software.

ABB compared to other quality measures

ABB can be used as a genotype-callability filter on top of other filters such as GATK VQSR, Fisher strand bias, genotype quality (GQ), Hardy-Weinberg Equilibrium (HWE), GIAB mappability scores etc. In order to validate the power of ABB as variant filter we used a variant call set to which all commonly used filter criteria had already been applied (see paragraph above).

<u>High callable sites provided by GIAB</u>

One exception is the GIAB high confidence regions (HCR) set provided by Genome in a Bottle (GIAB v3.3.2), which we did not apply. We therefore compared the performance of ABB and GIAB-HCR on identification of false positive variant calls.

GIAB-HCR provides a list of callable sites across the whole genome. Considering all exons of the autosomes (79,660,917bp included in the ABB model), GIAB classifies 75,442,680 sites as callable, leaving 4,218,237 sites as 'un-callable'. In comparison ABB classifies 46,396 sites as low or very low confidence (ABB >= 0.75), which we are for the sake of comparison to GIAB considering as 'un-callable' from here on.

Of the 46,396 sites labeled un-callable by ABB, 52% are classified as callable by GIAB, demonstrating that the two methods are not redundant and that systematic errors identified by ABB are not always caught by the GIAB model (see Sup. Fig. S3). We wondered if the 24,863 sites labeled un-callable by ABB but not by GIAB actually contain false positive variant calls. To this end, we interrogated the 40 false positive SNV calls of GATK/VQSR we confirmed as false by Sanger sequencing (out of the 209 sites evaluated by Sanger in total). Supp. Table 8 shows the classification of GIAB compared to the classification of ABB (split into the 4 ABB confidence

regions used throughout our study, i.e. ABB high, medium, low and very low confidence). 9 out of 40 (22.5%) false positive SNVs that were classified as callable positions by GIAB (i.e. GIAB fails to filter these false positive SNVs) were correctly identified as low or very low confidence by ABB. On the other hand, two false positives (5%) were correctly classified by GIAB, but not by ABB. Note that ABB identified more false positives (30 vs. 23 out of 40), while filtering substantially less sites across the whole exome than GIAB (40kb vs. 4MB).

Genotype quality (GQ)
Although we filtered calls with genotype quality below 20 (1 % error rate), we next interrogated if for the remaining variants, ABB correlated negatively with GQ, in order to check if the two filter scores were redundant. We split this analysis in two parts: (1) analysis of GQ and ABB in 10,000 SNPs randomly subsampled from 10 individuals analyzed in this project; and (2), correlation of GQ and ABB in 209 calls evaluated by Sanger sequencing. We did not find a correlation between GQ and ABB in the 10,000 SNPs randomly selected, showing an R^2 of 0.0022 (Supp. Fig. S4A). Additionally, we observed that ABB biased (ABB >= 0.9) and non-biased (ABB < 0.75) sites showed the same distribution of GQs, with the vast majority of variants reported by GATK having GQ >= 99 (Supp. Fig. S4B-C).

Secondly, no-correlation between ABB and GQ was observed for 209 evaluated by Sanger sequencing (R2 = -0.0045, Supp. Fig. S5A), supporting our findings for random SNPs. Moreover, the distribution of the GQ values in true positive and false positive calls was highly similar, again with most variants having GQ >= 99 (Supp. Fig. S5B-C). Our results indicate that GQ cannot be used to identify the type of systematic errors that the ABB model has been trained to find. (Supp. Fig. S5B-C).

Hardy-Weinberg Equilibrium (HWE)
HWE is a powerful variant filter for population-scale studies and frequent variants. However, HWE analysis requires large cohorts to gain the statistical power necessary to reliably filter false variant calls. Moreover, its power is decreased with rare variants with AF < 1% (Graffelman & Moreno, 2013; Huang et al., 2016). Considering these limitations, HWE cannot be applied in single case or family diagnostics, and is not suitable for *de novo* germline calls or somatic mutations detected by tumor-normal paired analysis. Furthermore, HWE has limited applicability for RVAS tests, as these tests by definition rely on rare and ultra-rare variants.

Nonetheless, we investigated the correlation of ABB and HWE filters for non-rare variants in a large cohort (i.e. in a suitable setting for HWE). To this end, we measured HWE in 9227 variant sites with a MAF > 1 % within a cohort of 893 samples from this project. We applied the HWE exact test and marked SNPs with p-value < 0.01 (Bonferroni corrected) as HWE biased. The Venn diagram (Supp. Fig. S6) shows a partial overlap of 30% of SNPs labeled as biased by ABB and/or HWE.

We next compared HWE and ABB on the 209 variants evaluated by Sanger using the same parameters as described above for all common variants. The HWE filter correctly removed 14 out of 40 false positive calls (35 %), while ABB labeled 21 out of 40 variants (52.5 %) as very-low confident sites (ABB >= 0.9) and 30 out of 40 (75 %) as low confident sites (ABB >= 0.75) (see Supp. Table S9). Although we again observed around 30 % of overlap between HWE and ABB classifications, HWE could

only detect one false call that slipped through the ABB filter, while ABB identified up to 17 FP calls (42.5 % of the total) that passed the HWE filter. On the other hand, HWE removed 27 out of the 134 true positive SNPs (~ 20.15 % of TP variants), while ABB removed 21 TP SNPs (~ 15.67 %) when using ABB >= 0.9 and 41 (30.60 %) when using ABB >= 0.75 as cutoff (Supp. Table S10).

Rare Variant Association Study (RVAS) for Chronic Lymphocytic Leukemia

Pre-filtration. To filter out potential false positive variant calls from case and control samples we used five statistical annotation scores at individual variant sites and/or across individuals: 1) genotype quality score with a threshold of 20 at individual site and a minimum average across individuals of 25, 2) minimum of 10 reads for read depth, 3) minimum of 80% call rate across case and control cohorts, 4) alternative allele frequency with thresholds at individual variant sites of 0.2 and minimum average across individuals of 0.25, and 5) Phred-scaled p-value using Fisher's exact test to detect strand bias of 200 and removed the worst 10%. In addition, we applied the Variant Quality Score Recalibration (VQRS) included in the GATK framework. VQSR uses machine-learning algorithms to learn from each dataset the annotation profiles of high confident variants and low confident variants, integrating information from multiple dimensions (https://gatkforums.broadinstitute.org/gatk/discussion/39/variant-quality-score-recalibration-vqsr). Finally, we removed variants that were outside the intersection of all exome enrichment kits (Agilent SureSelect 50Mb and 71Mb and Nimblegen SeqEz v3), or in regions that were recurrently under-covered in at least one kit.

Variant annotation. All variants were annotated using ANNOVAR. We removed variants not falling in exonic or splicing sites and variants overlapping segmental duplications (segdup identity score >= 90%). Furthermore, we annotated the functional impact of variants using Combined Annotation Dependent Depletion (CADD) score, which is a phred-like score ranging from 1 to 99, and removed likely non-damaging variants using a threshold of CADD<10. We further removed variants with minor allele frequency (MAF) greater than 0.5% in 1000 Genome Project (1000 Genomes Project Consortium, 2010) or in Exome Variant Server (EVS) ((National Heart, Lung, and Blood Institute Exome Sequencing Project, found at http://evs.gs.washington.edu/EVS/) databases. We also calculated the MAF for each variant in our local population using our "control" sample group (individuals belonging to different diseases but cancer-free, including Obsessive Compulsive Disorder, Intellectual Disability, Alopecia Areata, Fibromyalgia, Parkinson, Essential Tremor, Cystic Fibrosis, Spinocerebellar ataxia, Neuromyelitis Optica, Stroke, Ataxia, ChiariMalformation, Myasthenia, Progressive Encephalopathy, Immunodeficiency, and Vitiligo) and removed variants with local MAF>0.5%.

Sample-based QC. We used two different metrics to detect extreme outlier samples. We first removed samples with anomalous variant numbers, by discarding those outside the range (25th percentile - 3*interquartile range) and (75th percentile + 3*interquatile range). We further performed principal component analysis using synonymous variants across the whole exome that are not in linkage disequilibrium (with $r2<0.2$) and with a minor allele frequency greater than 1% and removed outliers

identified in the first two components. Finally, we obtained 437 CLL cases, 780 controls (non-cancer patients) and 127.298 variants, which passed all filter criteria.

Rare Variant Association Study. To infer candidate cancer risk genes in the CLL dataset we performed SKAT-O, Burden, MiST and KBAC association tests. SKAT-O and Burden tests are implemented in the R package SKAT (https://www.hsph.harvard.edu/skat/download/) version 1.3.0. The Null model for both tests was computed using the SKAT_Null_Model function with output set to dichotomous outcome (out_type= "D") and no sample adjustment (Adjustment=FALSE). For the SKAT-O we used the SKATBinary function with default parameters except for "method" that was set to "optimal.adj" (equivalent to SKAT-O method). As weights, we used Minor allele frequencies (MAF) of variants transformed with Get_Logistic_Weights. The burden test was performed using the same function (SKATBinary) and parameters, except for 'method', which was set to "Burden". To perform the MiST test, we used the standalone R package (version 1) available at CRAN repository: cran.r-project.org/web/packages/MiST/index.html. Specifically, we used the function logit.weight.test with all default parameters. For KBAC we used the R implementation (tigerwang.org/software/kbac) and the included function KbacTest with parameters alpha=2.5e-06, num.permutation=1000000, and with all other parameters set to default values.

Source of bias in CTDSP2 and CDC27 analysis

A large majority of chronic lymphocytic leukaemia patients harboured a variant in the gene CTDSP2, but most of these variants showed a significant deviation of AB (Supp. Fig. S7). In order to find the reason for this deviated AB and the observed false association between CLL and CTDSP2, we divided the controls into two groups depending on the capture method used (Agilent SureSelect or Nimblegen SeqEz). As shown in Supp. Fig. 8, control samples sequenced using any of the Agilent SureSelect kits harboured significantly more variants with deviating AB in CTDSP2 than samples sequenced using Nimblegen SeqEz enrichment (P-value = 0.01307 with Pearson's chi-squared test, focussing only on the intersected regions of all the kits). Therefore, we hypothesise that an issue with some capture probes of the Agilent SureSelect kits is causing the bias. Hence, a false association of CTDSP2 and CLL was obtained because all CLL samples were processed using Agilent SureSelect, while controls were mixed. ABB was able to identify these biased positions based on the recurrent observation of deviated AB in a subset of samples.

Performing the same analysis for CDC27, we did not observe any association between controls carrying vs. not carrying variants in this gene and the capture method (P-value = 1 and Supp. Fig. 10). Hence, issues with hybridization oligos cannot explain recurrent observation of variants with deviated AB. Literature search revealed that biases in the distribution of SNV allele balances in CDC27 could be explained by retroduplications affecting a subset of exons (Abyzov et al., 2013; Jia et al., 2012). Therefore, we performed coverage analysis of the exons/probes where we observed low and very low confidence ABB scores and recurrently deviated AB. In order to avoid enrichment-kit specific biases in coverage we performed this analysis in Agilent 71Mb samples only. We calculated coverage for 11 hybridization probes of interest (i.e. probes harbouring weird SNPs) using coordinates provided by the manufacturer. Additionally, we performed library size and GC-bias correction. Finally, we separated

these samples into 2 groups: samples with deviated AB as detected by ABB, and ones with no significant deviation. We compared normalised coverage between groups for each probe separately using Wilcoxon test and observed two regions differently covered (q-value = 0.0165 after FDR correction, see Supp. Fig. S11). In both probes carriers of variants with deviated AB had a median of ~2.5 fold coverage compared to ~2 fold in non-carriers, indicating an extra retrotransposon duplication (increased copy-number), which might harbour a base change compared to the source region. As this extra copy is not annotated in the reference genome reads (including potentially divergent bases) are mapping to the source region, creating abnormal allele balance patterns. This example demonstrates that ABB is able to identify systematic errors induced by un-annotated duplications (or more generally un-annotated repeat copies).

How to use and interpret ABB

As ABB scores are probabilities scaling between 0 and 1, they can be flexibly used in various approaches, without the need to apply scaling functions or hard thresholds (as for instance methods returning p-values such as HWE would require). If a hard threshold is desired we suggest ABB >= 0.9 (relaxed filter, only very likely systematic errors are removed) or ABB >=0.75 (strict filter removing likely and very likely systematic errors, at the cost of more true positives being removed).

However, we suggest using a combination of quality filters, including for instance GQ, FS, HWE, GIAB map-ability and/or specific filters for somatic or *de novo* mutation analysis and ABB. For large cohorts, we additionally use of VQSR and genotype callability rate of focal sites across the cohort as features. In combination with other filter methods we suggest to use an ABB threshold of >= 0.9 for filtering. If variant call precision is a priority (e.g. for detecting *de novo* or somatic mutation, and for RVAS) we suggest to use ABB >=0.75 as threshold.

ABB can be included as a feature in machine learning based classifiers instead of a combination of hard filters. For instance, we used ABB in the pathogenicity classifier eDiVA-Score (http://ediva.crg.eu, unpublished) as one feature of the random forest (RF) model. Here, ABB enables the RF to identify and lower the rank of systematic false calls, without applying a hard threshold.

References

Abyzov, A., Iskow, R., Gokcumen, O., Radke, D. W., Balasubramanian, S., Pei, B., … Gerstein, M. (2013). Analysis of variable retroduplications in human populations suggests coupling of retrotransposition to cell division. *Genome Research*, *23*(12), 2042–52. http://doi.org/10.1101/gr.154625.113

Chang, M. T., Asthana, S., Gao, S. P., Lee, B. H., Chapman, J. S., Kandoth, C., … Taylor, B. S. (2016). Identifying recurrent mutations in cancer reveals widespread lineage diversity and mutational specificity. *Nature Biotechnology*, *34*(2), 155–163. http://doi.org/10.1038/nbt.3391

Graffelman, J., & Moreno, V. (2013). The mid p-value in exact tests for Hardy-Weinberg equilibrium. *Statistical Applications in Genetics and Molecular Biology*, *12*(4), 433–48. http://doi.org/10.1515/sagmb-2012-0039

Huang, Z., Rustagi, N., Zhi, D., Cupples, A., Gibbs, R., Boerwinkle, E., & Yu, F. (2016). Hardy Weinberg Exact Test In Large Scale Variant Calling Quality Control. *bioRxiv*, 95521. http://doi.org/10.1101/095521

Jia, P., Li, F., Xia, J., Chen, H., Ji, H., Pao, W., & Zhao, Z. (2012). Consensus Rules in Variant Detection from Next-Generation Sequencing Data. *PLoS ONE*, *7*(6), e38470. http://doi.org/10.1371/journal.pone.0038470

Papaemmanuil, E., Rapado, I., Li, Y., Potter, N. E., Wedge, D. C., Tubio, J., … Campbell, P. J. (2014). RAG-mediated recombination is the predominant driver of oncogenic rearrangement in ETV6-RUNX1 acute lymphoblastic leukemia. *Nature Genetics*, *46*(2), 116–125. http://doi.org/10.1038/ng.2874

Puente, X. S., Beà, S., Valdés-Mas, R., Villamor, N., Gutiérrez-Abril, J., Martín-Subero, J. I., … Campo, E. (2015). Non-coding recurrent mutations in chronic lymphocytic leukaemia. *Nature*, *526*(7574), 519–524. http://doi.org/10.1038/nature14666

Tarpey, P. S., Behjati, S., Cooke, S. L., Van Loo, P., Wedge, D. C., Pillay, N., … Futreal, P. A. (2013). Frequent mutation of the major cartilage collagen gene COL2A1 in chondrosarcoma. *Nature Genetics*, *45*(8), 923–926. http://doi.org/10.1038/ng.2668

Supporting Figures

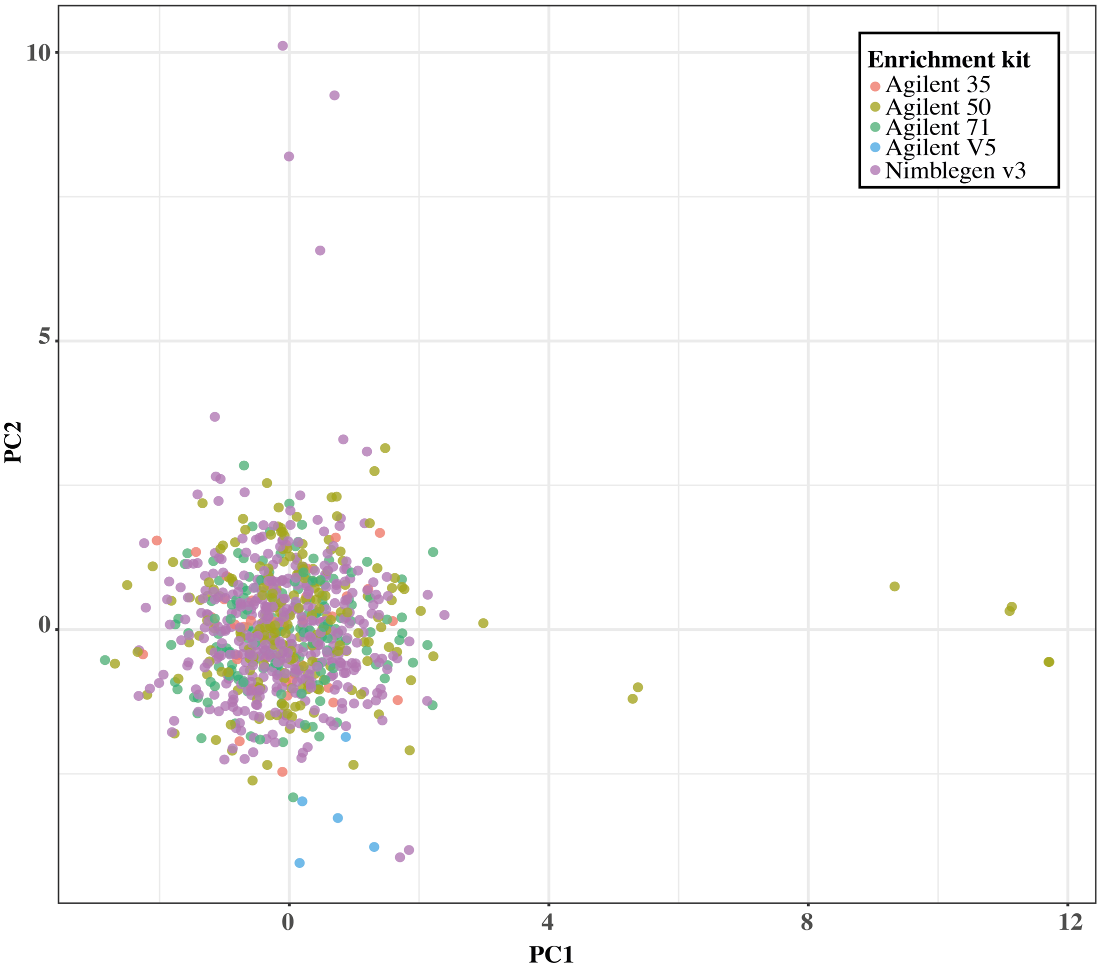

Supp. Fig. S1. Principal component analysis (PCA) based on all SNVs of all samples used in this study. Colors indicate different exome enrichment kits used to process samples. No bias between kits was observed when focusing on regions sufficiently covered (>= 10x) in all enrichment kits.

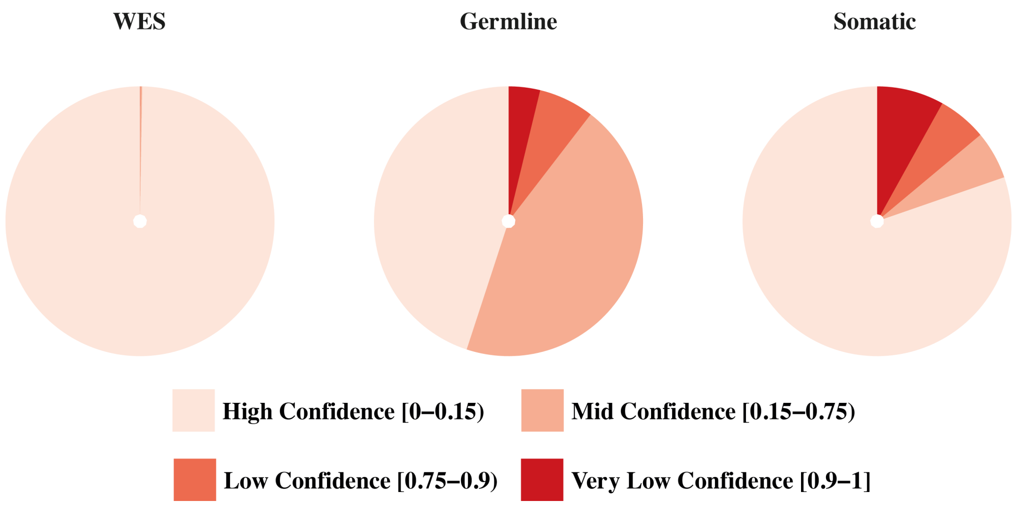

Supp. Fig. S2. Distribution of ABB callability confidence per analysis type, showing the proportion of high confidence (ABB < 0.15), mid confidence (0.15 ≤ ABB < 0.75), low confidence (0.75 ≤ ABB < 0.90) and very low confidence (ABB ≥ 0.9) positions for the whole exome (WES), for germline variants (Germline) and for somatic variants (Somatic).

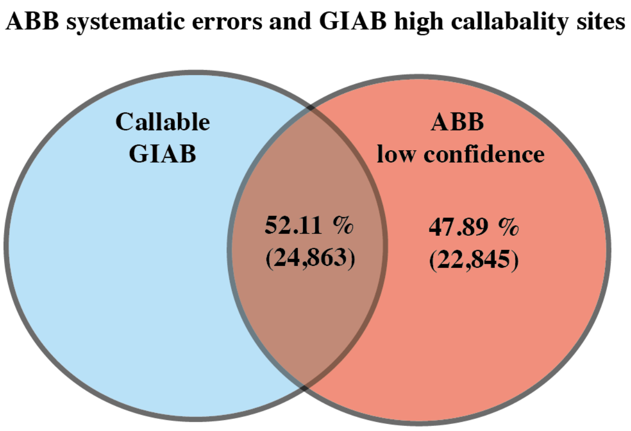

Supp. Fig. S3. Overlap of GIAB high confidence regions with ABB low and very low confidence sites (ABB >= 0.75). Only 47.89% of sites are equally labeled un-callable by both methods, while 52.11% of the sites labeled un-callable by ABB are considered callable by GIAB.

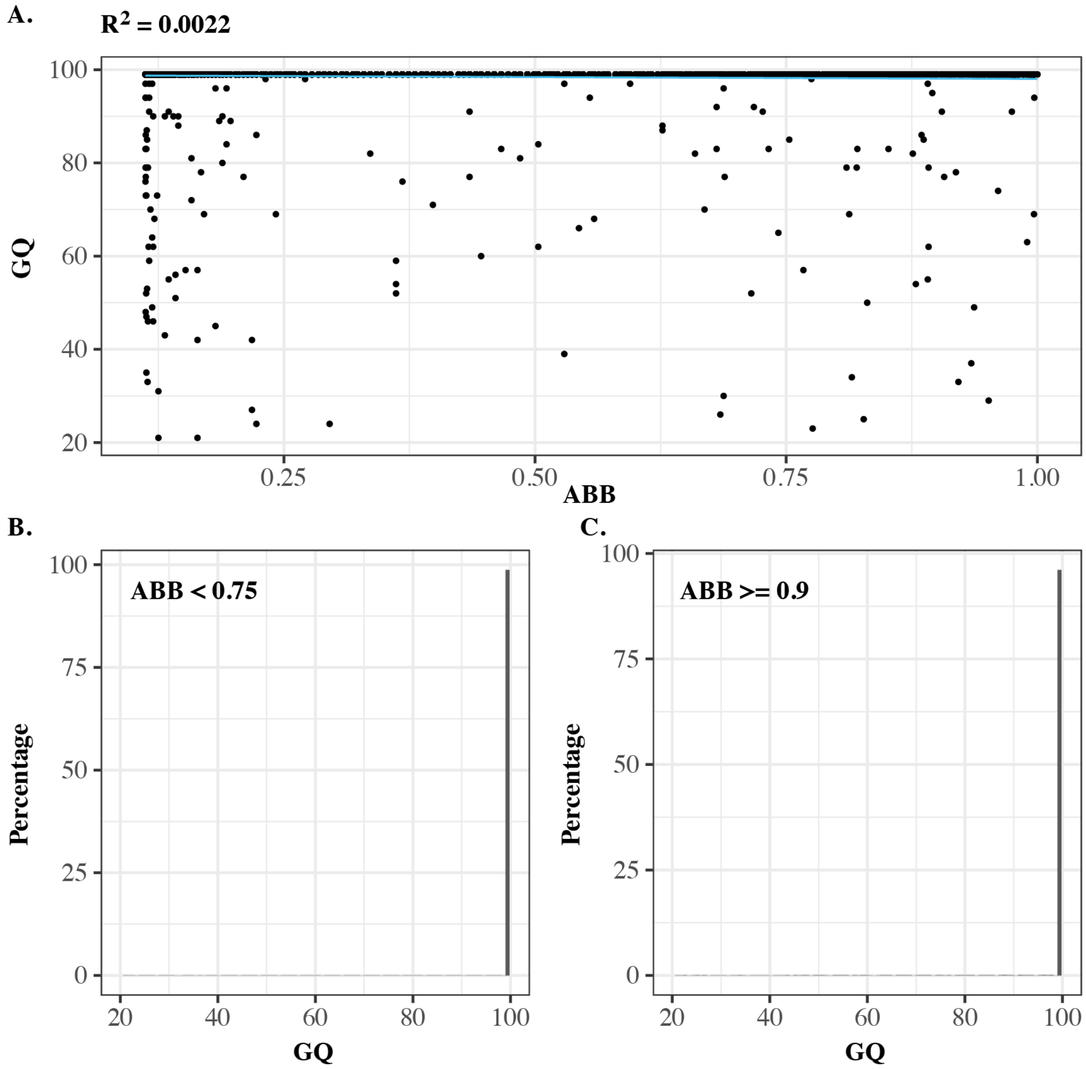

Supp. Fig. S4. GQ of 10,000 variant sites randomly subsampled from 10 samples. (A) Correlation plot, R2 = 0.002 (no correlation), (B) histogram of GQ for randomly subsampled variants with ABB <= 0.75, and (C) histogram of GQ for randomly subsampled variants with ABB >= 0.9.

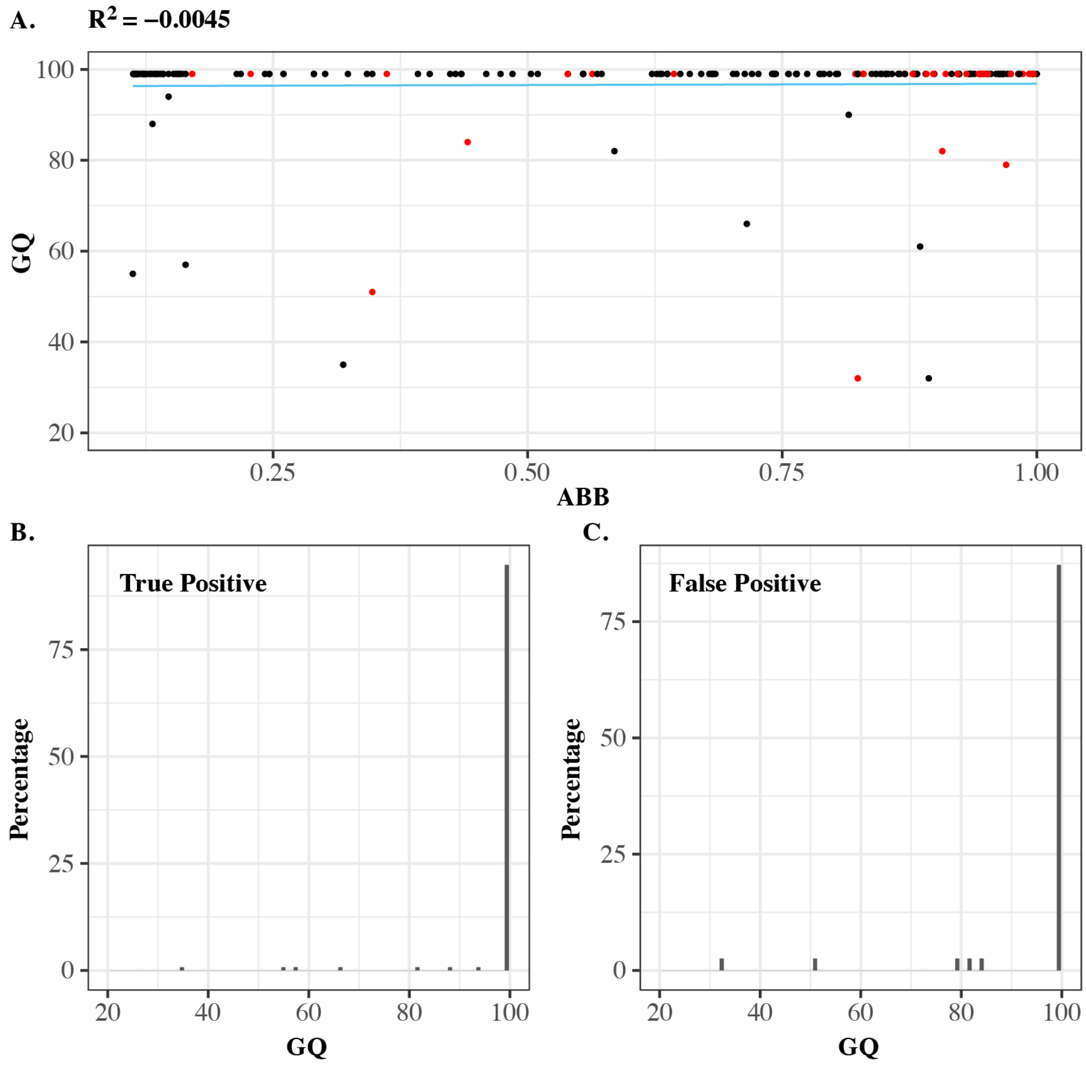

Fig. Supp. S5. GQ and ABB of 209 variants tested by Sanger sequencing. (A) Correlation plot with true positives in black, false positives in red (B) histogram of GQ true positive variants (C) histogram of GQ for false positive variants.

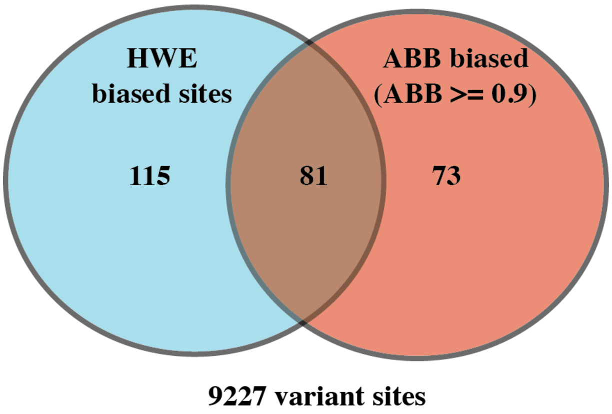

Supp. Fig. S6. Veen diagram of the sites filtered by Hardy-Weinberg Equilibrium test (HWE) and ABB (ABB >= 0.9). The analysis was performed in 9227 variant sites with population AF > 1 % in a cohort of 893 samples.

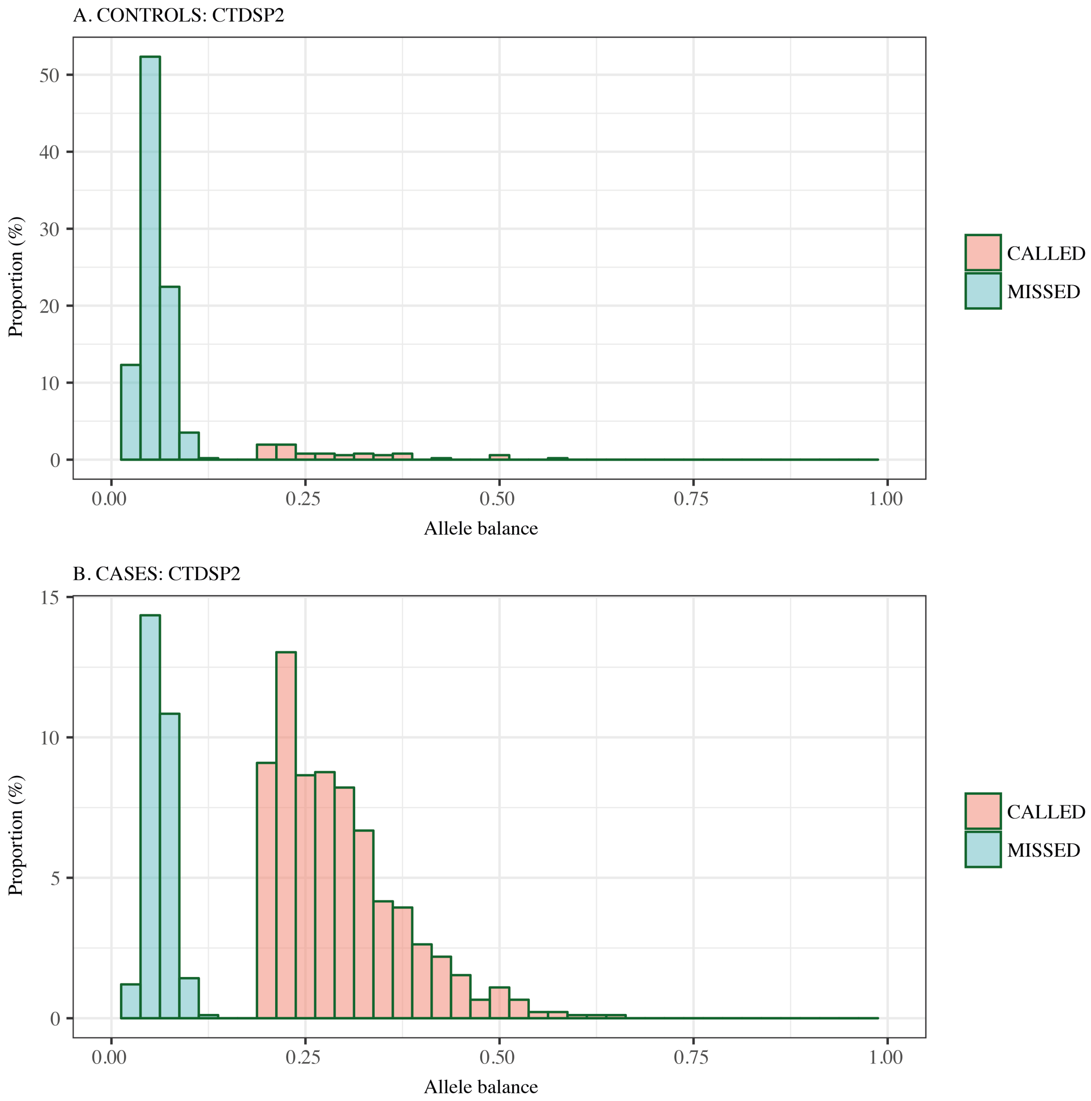

Supp. Fig. S7. Distribution of allele balances in the gene CTDSP2 for A) controls (Spanish non-cancer patients) and B) cases (ICGC-CLL cohort). Variants called by GATK *HaplotypeCaller* are labeled as "CALLED" and variants genotyped by GATK as homozygous reference but supporting more alternative alleles than expected by the zero-inflated beta distribution are labeled as "MISSED" calls.

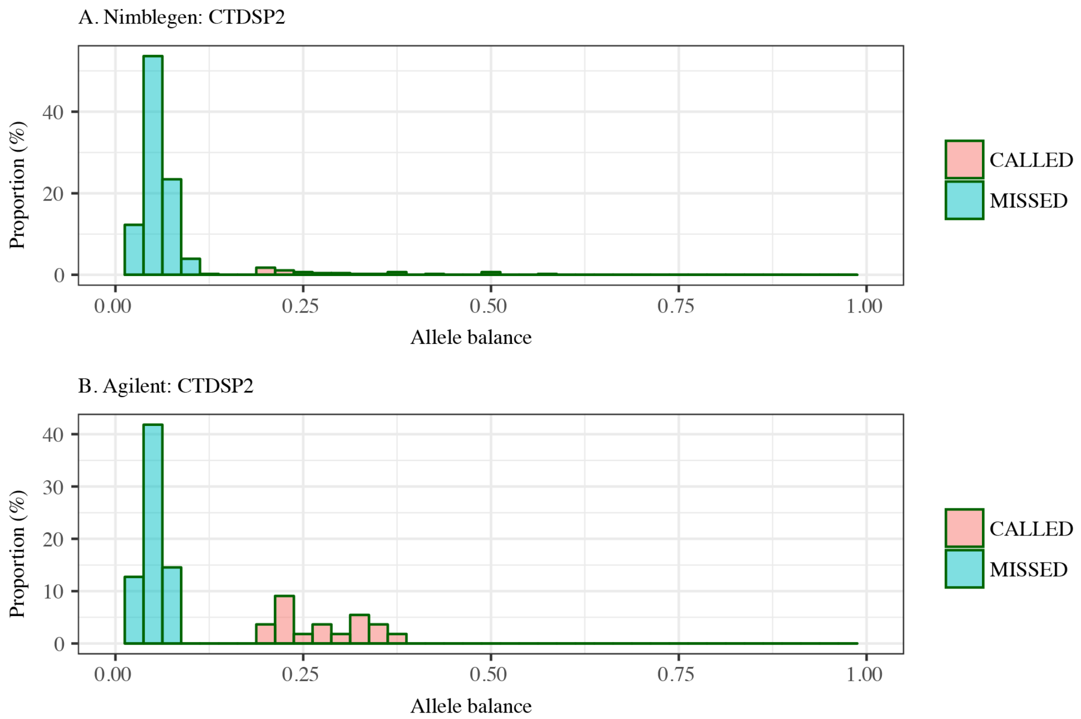

Supp. Fig. S8. Distribution of allele balances in the gene CTDSP2 for A) Nimblegen SeqEz, and B) Agilent SureSelect processed control samples. Variants called by GATK *HaplotypeCaller* are labeled as "CALLED" and variants genotyped by GATK as homozygous reference but supporting more alternative alleles than expected by the zero-inflated beta distribution are labeled as "MISSED" calls.

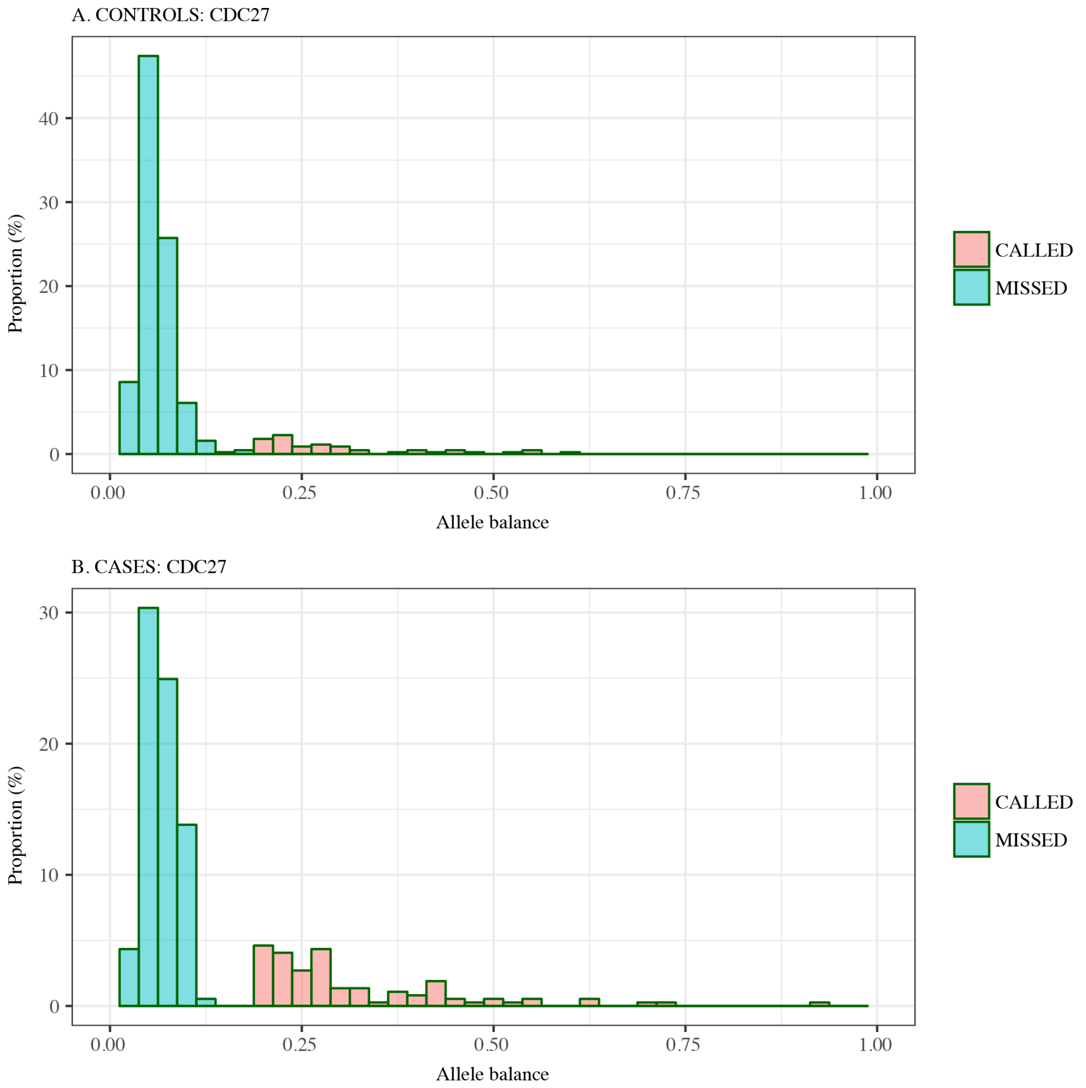

Supp. Fig. S9. Distribution of allele balances in the gene CDC27 for A) controls (Spanish non-cancer patients) and B) cases (ICGC-CLL cohort). Variants called by GATK *HaplotypeCaller* are labeled as “CALLED” and variants genotyped by GATK as homozygous reference but supporting more alternative alleles than expected by the zero-inflated beta distribution are labeled as “MISSED” calls.

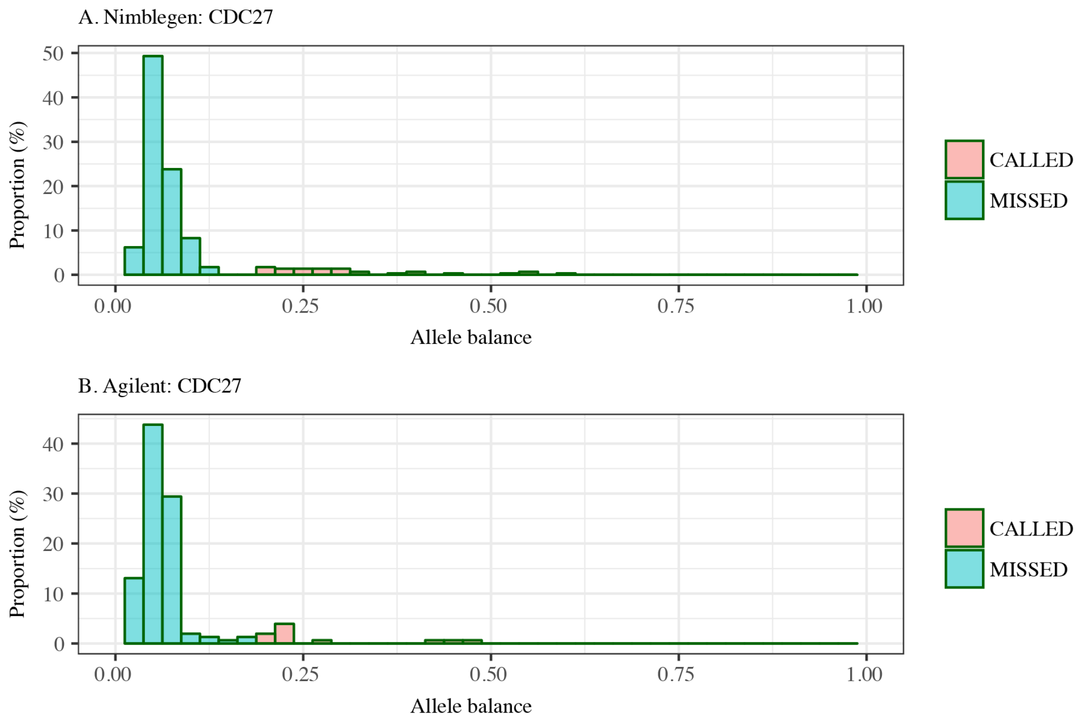

Supp. Fig. S10. Distribution of allele balances in the gene CDC27 for A) Nimblegen SeqEz, and B) Agilent SureSelect processed control samples. Variants called by GATK *HaplotypeCaller* are labeled as “CALLED” and variants genotyped by GATK as homozygous reference but supporting more alternative alleles than expected by the zero-inflated beta distribution are labeled as “MISSED” calls.

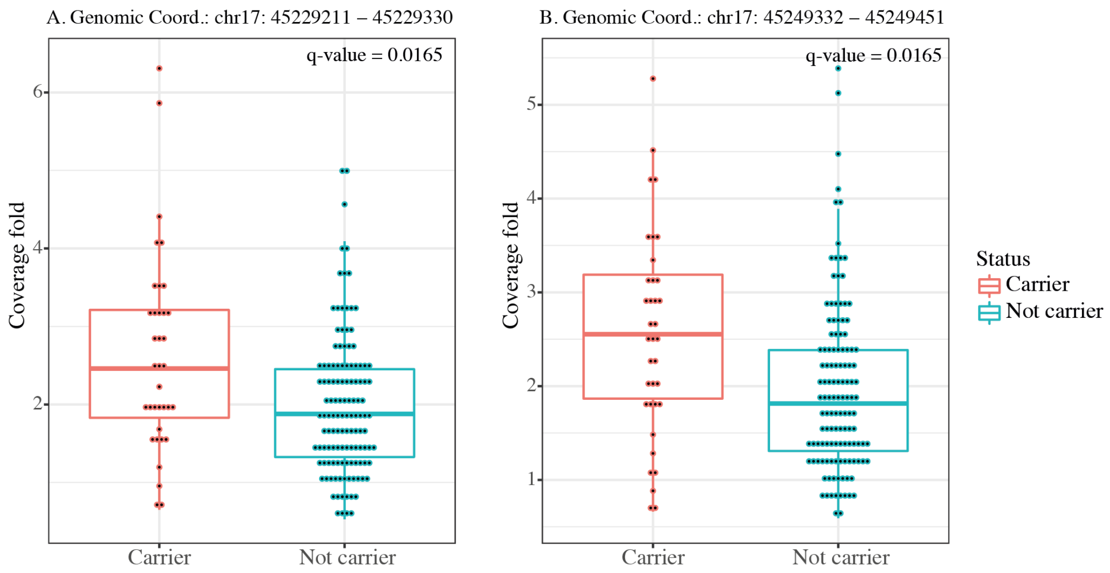

Supp. Fig. S11. GC- and library size- normalized and median-corrected coverage of 2 exons (A and B) with specified coordinates from Agilent 71MB kit, which harbor SNPs with unusual B-allele frequencies detected by ABB in the group 'Carriers', but noen in the group "Not carriers). We observed a significant difference in median coverage between groups, indicating an extra retroduplication copy in the Carrier group.

Supporting Tables

Supp. Table S1. Sample information for 1197 germline samples used for training or evaluation of the ABB model.

Supp. Table S2. Sample information for 200 CLL tumor samples used in the evaluation of ABB as a quality filter for somatic variant calls.

Supp. Table S3. Primer designs for Sanger sequencing.

Supp. Table S4. Performance of LR1 in the training, testing, and evaluation set.

Supp. Table S5. Contingency table for repetitive element fraction in very low and high confidence sites.

Supp. Table S6. Overlap of validated somatic variants from three studies (Tarpey et al., 2013; Papaemmanuil et al., 2014) and known cancer driver mutations hotspots (Chang et al., 2016) with positions labeled as systematic errors by ABB.

Supp. Table S7. Results of Sanger sequencing validation for 209 random sites.

Supp. Table S8. Classification of false positive SNV sites (as evaluated by Sanger sequencing) by GIAB and ABB. Sites are grouped by ABB confidence levels and GIAB classification. GIAB correctly classified 23 out of 40 and ABB 30 out of 40 false positives. 9/40 false positives are only found by ABB (shown in bold), 2 out of 40 false positives are only found by GIAB.

Supp. Table S9. Performance of ABB score and Hardy-Weinberg equilibrium filter on Sanger-identified false positive calls.

Supp. Table S10. Performance of ABB score and Hardy-Weinberg equilibrium filter on Sanger-validated true positive calls.

Supp. Table S11. Sanger validation of candidate disease variants found in various studies, including true positive and false positive SNV calls. Red: very low-confidence sites (ABB >= 0.9), orange: low confidence sites (0.75 <= ABB < 0.9), yellow: mid-confidence sites (0.15 <= ABB < 0.75), and green: high-confidence sites (ABB < 0.15).

Supp. Table S12. List of variant sites labeled as significant in the ABB association test based on Missed-Called ratio (FDR) obtained from the missed-called ratio test (methods).

Supp. Table S13. Genes labeled as prone to false positive associations based on three test (methods): Missed-Called ratio (FDR) obtained from the missed-called ratio test; Association re-genotyped (FDR) from association chi square test between cases and control including re-genotyped variants; Association ABB (FDR) association analysis with chi square test removing prone to significant variant sites.
